# Neopterin as a Biomarker of Cellular Immune Response in Renal Allograft Rejection Subtypes: Linking Cytokines and Immune Cells to Improve Diagnostic and Therapeutic Approaches

**DOI:** 10.3390/biomedicines14040832

**Published:** 2026-04-06

**Authors:** Ravi Dhital, Mukut Minz, Ranjana Walker Minz, Shashi Anand, Ritambhra Nada, Sarbpreet Singh, Deepesh B. Kenwar, Ashish Sharma

**Affiliations:** 1 Center for Childhood Cancer Research, Nationwide Children’s Hospital, Columbus, OH 43215, USA; ravi.dhital@nationwidechildrens.org; 2 Department of Renal Transplant Surgery, Post Graduate Institute of Medical Education and Research, Chandigarh 160012, India; mukutminz@hotmail.com (M.M.); drsarbpreet@yahoo.com (S.S.); deepesh.doc@gmail.com (D.B.K.); ashish_chd@hotmail.com (A.S.); 3 Department of Immunopathology, Post Graduate Institute of Medical Education and Research, Chandigarh 160012, India; sanand312@gmail.com; 4 Department of Histopathology, Post Graduate Institute of Medical Education and Research, Chandigarh 160012, India; ritamduseja@yahoo.com

**Keywords:** renal transplantation, acute allograft rejection, neopterin, cytokines, T cells, NK cells

## Abstract

**Background**: Renal allograft rejection remains a major challenge in transplantation. Current diagnostic approaches, including biopsies, are invasive and may fail to detect subclinical immune activation, potentially contributing to progressive graft dysfunction. Reliable, non-invasive biomarkers capable of monitoring immune activation and distinguishing rejection phenotypes are therefore needed. **Methods**: In this retrospective study, we evaluated serum neopterin as a biomarker of immune activation and graft status over 12 months following transplantation. Associations between neopterin levels and immune parameters, including natural killer (NK)-to-CD3^+^CD16/CD56^+^ T cell ratios, cytokines (IFN-γ and IL-10), and CD4^+^CD25^+^FoxP3^+^ T cell frequencies, were assessed. A total of 211 first renal allograft recipients were followed longitudinally, including patients with acute rejection (AR) and matched stable graft (SG) recipients. Serum neopterin was quantified by enzyme immunoassay, and immunophenotyping, mRNA expression, and cytokine profiling were performed on peripheral blood samples. **Results**: Serum neopterin levels were significantly elevated in AR compared to SG recipients, with a threshold of 57 nmol/L distinguishing AR with 81% sensitivity and 80% specificity. While IFN-γ demonstrated higher diagnostic performance in cross-sectional analysis, neopterin showed a more sustained elevation over time, remaining increased in AR recipients even at later post-transplant time points. Neopterin correlated positively with IFN-γ, but not IL-10, and inversely with CD4^+^CD25^+^FoxP3^+^ T cell frequency. NK cells were enriched during rejection, whereas CD3^+^CD16/CD56^+^ T cells were more prominent in graft stability. The NK-to-CD3^+^CD16/CD56^+^ T cell ratio was highest during acute cellular rejection. **Conclusions**: Neopterin reflects Th1-associated immune activation in renal allograft recipients and provides a temporally stable, non-invasive marker of immune status. Although it does not outperform IFN-γ levels at the time of rejection, its stability and sustained elevation suggest a complementary role in longitudinal monitoring. Integration of neopterin with immune parameters, including cytokine profiles and cellular subsets, may enhance the assessment of graft immunological status and support clinical decision-making.

## 1. Introduction

Neopterin [2-amino-4-hydroxyl-6-(D-erythro-1′, 2′, 3′-trihydroxypropyl)-pteridine] is a pyrazino-pyrimidine derivative produced during interferon-gamma (IFN-γ)-induced guanosine triphosphate (GTP) metabolism in activated monocytes and macrophages [1,2]. Since its discovery in 1889, neopterin has been a well-established marker of cellular immune activation and is detectable in multiple biological fluids, including serum, urine, cerebrospinal fluid, and synovial fluid [3,4,5,6,7,8]. Neopterin release precedes T cell proliferation and may be detected in serum prior to the development of antigen-specific antibody responses [9], highlighting its utility as an early indicator of immune activation.

Elevated neopterin levels have been reported across a wide spectrum of inflammatory, infectious, autoimmune and malignant conditions, typically reflecting Th1-skewed immune response [10,11]. In transplantation, neopterin has been associated with immune activation following hematopoietic stem cell transplantation and the development of graft-versus-host disease (GVHD) [12,13,14]. In solid organ transplantation, stable neopterin levels are generally observed in uncomplicated post-transplant courses, whereas elevated levels are associated with rejection or infections [15,16,17,18].

Despite this, prior studies in kidney transplantation have predominantly examined neopterin as a general marker of rejection, without resolving its relationship to specific rejection phenotypes such as T cell-mediated rejection (TCR), antibody-mediated rejection (ABMR), or mixed rejection. Given that neopterin reflects macrophages and monocyte-driven immune activation, its levels are likely shaped by interactions with key immune subsets, including NK cells, NKT cells, and regulatory T cells (Tregs), as well as cytokine networks such IFN-γ and IL-10.

NK cells, implicated in ABMR [19,20], and NKT cells, associated with immune regulation and tolerance [21], may modulate neopterin production through crosstalk with monocytes and macrophages. Similarly, the balance between IFN-γ and IL-10 is critical in determining the inflammatory milieu, with IFN-γ promoting rejection and IL-10 supporting immune regulation [22,23]. As major producers of IL-10 [24], Tregs may further influence neopterin dynamics through immunomodulatory effects.

Here, we investigated the relationship between serum neopterin levels and immune cell composition, cytokine balance, and rejection phenotype. We hypothesized that neopterin reflects the balance between pro- and anti-inflammatory immune responses and may discriminate between rejection subtypes. Our goal was to evaluate neopterin as a clinically accessible biomarker that, alone or in combination with immune parameters, could support improved diagnosis and management of renal allograft rejection.

## 2. Methods

### 2.1. Patients and Controls

This study represents a retrospective analysis of a prospectively followed cohort of renal transplant recipients at the Department of Renal Transplant Surgery, Post Graduate Institute of Medical Education and Research (PGIMER) [25,26,27,28,29,30,31]. The cohort included 211 consecutive adult patients undergoing primary renal transplantation to minimize selection bias. Inclusion criteria comprised first renal allograft recipients with available longitudinal clinical data and archived serum samples, and negative serology for HIV, HBsAg and HCV at the time of transplantation. Exclusion criteria included multi-organ transplantation, prior transplant history, incomplete clinical follow-up and evidence of active systemic infection at the time of sample collection, where applicable.

Participants were followed for 12 months post-transplantation. Demographic and clinical data were collected, and blood samples were obtained pre-transplant and at 1, 3, 6, and 12 months post-transplantation. Serum samples were processed under standardized conditions and archived for subsequent neopterin quantification. Acute allograft rejection episodes were identified based on clinically indicated biopsies. Blood samples from living donors prior to nephrectomy served as healthy controls. Potential confounders were addressed by monitoring transplant-associated infections, adjusting immunosuppressive therapy based on trough levels, and assessing renal functions using serum and urinary creatinine measurements.

The study was approved by the institutional ethics committee, and written informed consent was obtained from all participants. Secondary analysis of archived samples was conducted under the category of secondary research utilizing previously collected specimens (Endorsement No. 8768/1TRG-PG/11/5833, 2 May 2012). Flow cytometry, cytokine and mRNA expression data were derived from the parent study dataset. Acute allograft rejection cases (*n* = 38) from 35 recipients were identified, and a control cohort of 38 recipients with stable grafts (SG) was matched for age and sex.

### 2.2. Serum Neopterin Measurement

Serum neopterin levels were measured using a commercially available ELISA Kit (IBL International GMBH, Hamburg, Germany) according to the manufacturer’s instructions. All samples were assayed in duplicate, with <10% variance between replicate measurements. Positive and negative controls were included in each run and fell within the manufacturer’s recommended range. Neopterin concentrations are reported as nanomoles per liter (nmol/L) of blood.

### 2.3. Peripheral Immunophenotyping by Flow Cytometry

PBMCs were isolated from EDTA-treated blood by density gradient centrifugation (Histopaque-1077, Sigma, St. Louis, MO, USA). Approximately 2 × 10^6^ cells were stained with fluorochrome-conjugated monoclonal antibodies against CD45, CD11b, CD3, CD4, CD8, CD16, CD56, CD25 and FoxP3 (Table S1). Surface staining was performed at room temperature for 45 min, followed by fixation (4% paraformaldehyde) and permeabilization using FoxP3 buffer (BD Bioscience, Milpitas, CA, USA) for intracellular staining.

Data were acquired on BD FACS Aria II or BD FACS Canto II cytometers and analyzed using BD FACSDiva v6.2 or FlowJo software v10.10.0. Lymphocytes were identified on forward and side scatter, followed by singlet gating and CD45^+^CD11b^−^ population selection. T cells were defined as CD3^+^, with CD4^+^ and CD8^+^ subsets further delineated. A regulatory T cell-enriched population was defined as CD4^+^CD25^+^FoxP3^+^ cells. NK cells were defined as CD3^−^CD16^+^/CD56^+^, and CD3^+^CD16^+^/CD56^+^ T cells were analyzed as a compositive population. Representative gating strategy and antibody panels are provided in Supplementary Figure S1 and Table S1.

Instrument performance was standardized using calibration beads, and compensation was performed using single-stained controls. Unstained cells were used to assess background fluorescence, and isotype controls were included for intracellular FOXP3 staining. Antibody titrations were optimized using PBMCs from healthy donors. Samples were processed under uniform conditions and analyzed using consistent gating templates to minimize inter-assay variability.

### 2.4. mRNA Gene Expression

Total RNA was extracted from EDTA-treated blood using the QIAamp RNA Blood Mini Kit (Qiagen, Germantown, MD, USA). First-strand cDNA was synthesized from 200 ng RNA using a mix of oligo (dT) and random hexamer primers with the RevertAid first-strand cDNA synthesis Kit (Thermofisher Scientific, Waltham, MA, USA). cDNA purity was assessed by A260/A280 ratio. Relative gene expression of *CD80* and *FOXP3* was quantified using real-time PCR (Roche Molecular System, Pleasanton, CA, USA) with SYBR Green and gene-specific primers. The amplification protocol consisted of 95 °C for 10 min, followed by 45 cycles of 95 °C for 10 s, 60 °C for 15 s, and 72 °C for 20 s. Relative expression level (fold change) of genes of interest (GOI), normalized to housekeeping gene (HKG) *β-actin*, was calculated using the formula 2^−ΔΔCT^ = ΔCT_Post-transplant_ − ΔCT_pre-transplant_ where ΔCT_Post-transplant_ = (CT_GOI_ − CT_HKG_)_post-transplant_ and ΔCT_Pre-transplant_ = (CT_GOI_ − CT_HKG_)_pre-transplant_.

### 2.5. Serum IFN-γ and IL-10 Quantification

Serum cytokine levels were measured using the BD^TM^ Cytometric Bead Array (CBA) Human Th1/Th2/Th17 Cytokine Kit (BD Bioscience, CA, USA). This assay enables simultaneous detection of multiple cytokines. Capture beads for IL-2, IL-4, IL-6, IL-10, TNF-α, INF-γ and IL-17A were incubated with standards or samples and detected using PE-conjugated antibodies. Fluorescence intensity was acquired on a BD FACS Aria II and analyzed using BD FACSDiva v6.2 and FCAP Array software v3.0. Cytokine concentrations are reported as picograms per milliliter (pg/mL) of blood.

### 2.6. Statistics

Data are presented as mean ± standard error of the mean (SEM). Normality was assessed using the Shapiro–Wilk test (two-sided, α = 0.05). For normally distributed data, comparisons between two groups were performed using two-tailed Welch’s *t*-test. For comparisons involving more than two groups, ordinary one-way ANOVA with Tukey’s multiple-comparison test was applied. For data that did not meet normality assumptions, non-parametric tests were used. Two-group comparisons were performed using the Mann–Whitney U test, and multi-group comparisons were analyzed using the Kruskal–Wallis test (or Friedman test for paired data) followed by Dunn’s multiple-comparison correction.

For analyses involving small cohorts (ABMR, *n* = 8; mixed rejection, *n* = 9), post hoc effect size (Cohen’s f for ANOVA-based analyses) and achieved power were calculated using G*Power v3.1.9.7. Correlations between neopterin and demographic variables were evaluated using Pearson’s correlation coefficient. Sensitivity and specificity were evaluated using Receiver Operating Characteristic (ROC) curves.

All statistical analyses were performed using GraphPad Prism v10.6.0 (San Diego, CA, USA), and *p*-values < 0.05 were considered statistically significant.

## 3. Results

### 3.1. Cases and Control

Among 211 first kidney allograft recipients, 35 patients (16.58%) experienced 38 biopsy-confirmed acute rejection episodes within the first-year (acute rejection; AR group). From the remaining 176 recipients without rejection, 38 were matched for age, sex, HLA mismatch, immunosuppression, and donor type to form the stable graft (SG) group. Thirty-five age- and sex-matched donors served as healthy controls (HCs). Baseline characteristics are summarized in Table 1.

Recipients received induction therapy with rabbit antithymocyte globulin (ATG; total dose 3.5 mg/kg administered in three doses starting 12 h before transplantation) or anti-IL-2 receptor therapy (total dose 20 mg administered in two doses, beginning 30 min before transplantation). Maintenance immunosuppression included tacrolimus (TAC) or cyclosporine-A (CSA), mycophenolate mofetil (MMF), and corticosteroids, with dose adjustments targeting trough levels of 5–7 ng/mL for TAC and 150–200 ng/mL for CSA.

### 3.2. Rejection Characteristics

Acute allograft rejection episodes were classified according to the Banff criteria [32] into cellular (ACR, *n* = 21), antibody-mediated (ABMR, *n* = 8), and mixed (*n* = 9) rejection. The mean time to rejection was 34.8 days for ACR (range, 3–194), 44.6 days for ABMR (range, 3–190), and 26.9 days for mixed rejection (range, 2–93) post-transplantation.

Cellular rejection was initially treated with intravenous methylprednisolone (500 mg/day for three days), with steroid-resistant cases receiving ATG for 10–14 days. Mixed rejection was managed similarly, followed by ABMR-directed therapy, including plasmapheresis, intravenous immunoglobulin (IvIg), and rituximab.

### 3.3. Serum Neopterin Levels in ESRD Patients

Serum neopterin levels were measured across 213 samples at multiple time points: pre-transplant (*n* = 70), at acute rejection (*n* = 38), 12 months post-transplant (*n* = 70), and in SG controls at 1-month post-transplant (*n* = 35).

**Figure 1:**
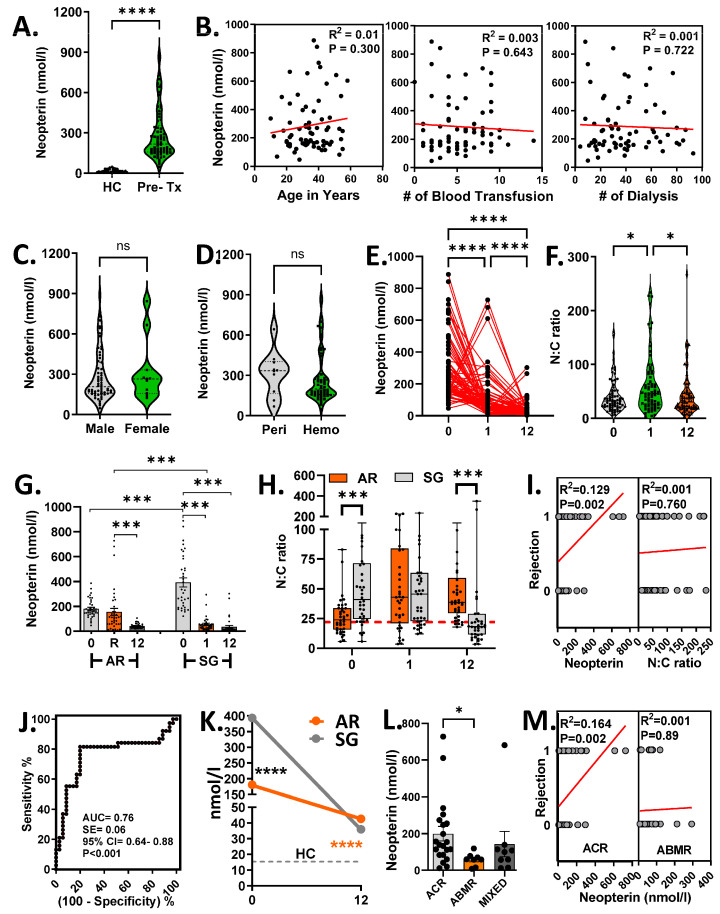
Serum neopterin levels and neopterin-to-creatinine (N:C) ratio in renal transplant recipients. A total of 211 first-time renal allograft recipients were prospectively followed for 12 months. Recipients (*n* = 35) with 38 rejection episodes were classified as AR. A cohort of matched recipients (*n* = 38) without rejection served as SG. Age- and sex-matched donors (*n* = 35) were included as HC. Peripheral blood samples were collected, with serum separated and archived at −80 °C until analysis. Serum neopterin concentrations were quantified using ELISA, and the N:C ratio was calculated for normalization. (**A**) Serum neopterin levels between uremic patients (pre- Tx) and healthy controls (HC). (**B**) Correlation between serum neopterin levels and age of the patients, number of blood transfusions and dialysis. (**C**) Pre-transplant neopterin levels between males and females. (**D**) Variation in pre-transplant neopterin levels between the types of dialysis. Peri = peritoneal; Hemo = hemodialysis. (**E**,**F**) Post-transplant changes in the serum neopterin levels (**E**) and N:C ratio (**F**) of all the transplant recipients. (**G**,**H**) Differences in the neopterin levels (**G**) and N:C ratio (**H**) between AR and SG groups. (**I**) Spearman correlation between neopterin/N:C ratio and rejection. 0 = no rejection; 1 = presence of rejection. (**J**) Receiver Operating Characteristic (ROC) curve for serum neopterin. A cut-off of 57 nmol/L was selected to have the best sensitivity and specificity to separate AR from SG. AUC = area under curve; SE = standard error; CI = confidence interval. (**K**) Serum neopterin levels between AR and SG cohorts at pre-transplant (0) and 12 months post-transplantation (12). (**L**) Differences in the serum neopterin levels between ACR, ABMR and mixed. (**M**) Spearman correlation between neopterin and rejection (ACR/ABMR). 0 = no rejection; 1 = presence of rejection. Analysis: Mann–Whitney U test (**A**,**C**,**D**,**H**,**K**); Kruskal–Wallis test with Dunn’s multiple comparison test (**E**–**G**,**L**). ns, not significant; * *p* < 0.05; *** *p* < 0.001; **** *p* < 0.0001.

Pre-transplant uremic patients exhibited significantly higher serum neopterin levels compared to healthy controls (288.5 ± 23.05 nmol/L vs. 15.36 ± 1.851 nmol/L, *p* < 0.001) (Figure 1A). Neopterin levels did not correlate with age, number of blood transfusions, or dialysis sessions, and were not significantly influenced by sex or dialysis modality (Figure 1B–F), suggesting that elevated pre-transplant neopterin levels primarily reflect the uremic state.

### 3.4. Post-Transplant Neopterin Dynamics

Following transplantation, serum neopterin levels decreased significantly at 1 month (108.1 ± 16.45 nmol/L, *p* < 0.001) and 12 months (39.12 ± 5.73 nmol/L, *p* < 0.001) (Figure 1E). This decline resulted in a significant increase in the neopterin-to-creatinine (N:C) ratio at 1 month (Figure 1F).

In SG recipients, neopterin levels decreased markedly at 1 month compared to baseline (393.3 ± 36.61 nmol/L vs. 55.86 ± 9.596 nmol/L, *p* < 0.001). In contrast, AR recipients showed no significant change at the time of rejection compared to baseline (156.2 ± 28.34 nmol/L vs. 183.7 ± 13.09 nmol/L, *p* = 0.577) (Figure 1G). Neopterin levels in AR were significantly higher than in SG at 1-month post-transplantation.

Although the pre-transplant N:C ratio was higher in the SG group, no significant differences were observed between groups post-transplant (Figure 1H). These findings suggests that neopterin is a more reliable marker than the N:C ratio for distinguishing rejection from stable graft function (Figure 1I).

Receiver Operating Characteristic (ROC) analysis identified 57 nmol/L as the optimal cut-off to distinguish AR from SG, with 81.5% sensitivity (95% CI = 65.67–92.26) and 80% specificity (95% CI = 63.06–91.56), yielding 83.7% positive predictive value (PPV), 80.5% negative predictive value (NPV), and an overall diagnostic accuracy of 82.1% (Figure 1J).

### 3.5. Late Post-Transplant Neopterin Levels

At 12 months post-transplant, both AR and SG groups demonstrated significant reductions in neopterin levels relative to baseline (Figure 1K). Neopterin levels in SG recipients approached those of healthy controls (36.02 ± 11.55 nmol/L vs. 15.36 ± 1.85 nmol/L, *p* = 0.08), whereas AR recipients maintained levels approximately threefold higher than controls (42.68 ± 3.29 nmol/L vs. 15.36 ± 1.85 nmol/L, *p* < 0.001).

### 3.6. Neopterin Levels Across Rejection Types

Serum neopterin levels differed among rejection types, with the highest levels observed in ACR compared to ABMR (199.2 ± 39.71 nmol/L vs. 58.59 ± 12.42 nmol/L, *p* = 0.0410) (Figure 1L, Cohen’s f = 0.45, G*power = 0.68). However, the neopterin levels did not significantly distinguish mixed rejection from other groups (Figure 1M). These findings support the potential of neopterin as a non-invasive marker to differentiate ACR from ABMR.

### 3.7. Correlation of Pro- and Anti-Inflammatory Responses with Neopterin

**Figure 2:**
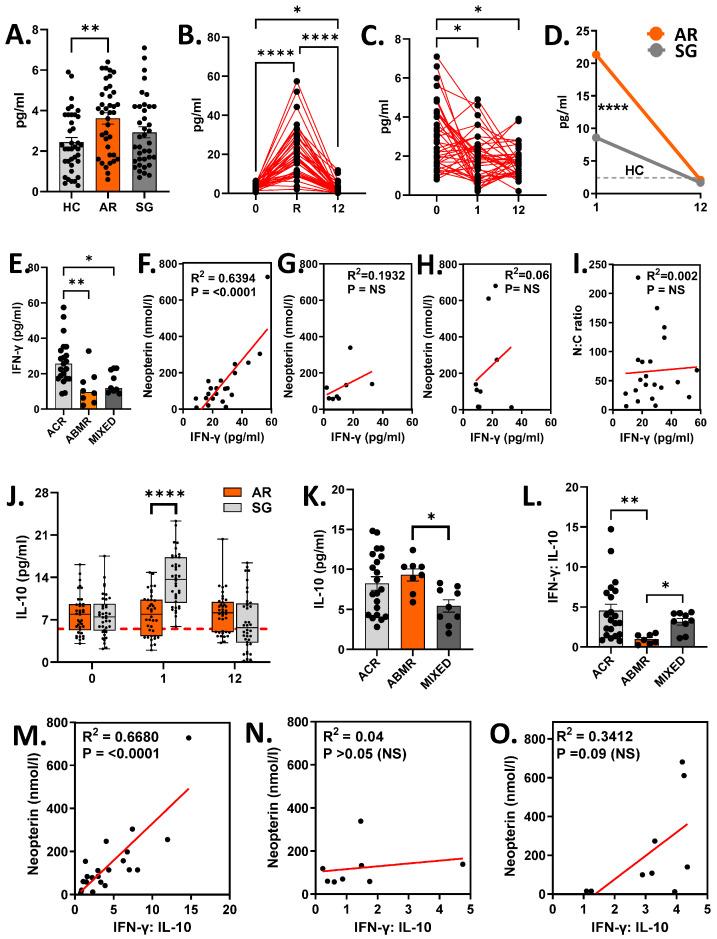
Correlation of serum quantity of IFN-γ/IL-10 with serum neopterin levels. Peripheral blood samples from transplant recipients and healthy controls were collected, with serum isolated and stored at −80 °C until analysis. Samples were thawed gradually on ice, gently mixed, and briefly centrifuged. Serum neopterin levels were quantified using ELISA, while cytokine concentrations were measured using multiplex bead-based immunoassay. (**A**) Serum IFN-γ levels (pg/mL) among transplant recipients. (**B**,**C**) Changes in the serum IFN-γ levels in AR (**B**) and SG (**C**) recipients at pre-transplant (0) and at 1 (1) and 12 months (12) post-transplantation. (**D**) Differences in the serum IFN-γ levels between AR and SG recipients at the time of rejection (1) and stable allograft functions (12). (**E**) IFN-γ levels in ACR, ABMR and mixed cohorts at the time of rejection. (**F**–**H**) Spearman correlation between IFN-γ and neopterin in ACR (**F**), ABMR (**G**) and mixed rejection (**H**). (**I**) Spearman correlation between IFN-γ and N:C ratio in ACR recipients. (**J**) Differences in the serum IL-10 levels between AR and SG recipients. (**K**) Serum IL-10 levels in ACR, ABMR and mixed rejection. (**L**) IFN-γ:IL-10 ratio in ACR, ABMR and mixed rejection. (**M**–**O**) Spearman correlation between IFN-γ:IL-10 ratio and neopterin in ACR (**M**), ABMR (**N**) and mixed (**O**) rejection. Analysis: Welch’s *t* test (**J**); Mann–Whitney U test (**D**); Kruskal–Wallis test with Dunn’s multiple comparison test (**A**,**L**); Friedman test with Dunn’s multiple comparison test (**B**,**C**); ordinary one-way ANOVA with Tukey’s multiple comparison test (**E**,**K**). NS, not significant; * *p* < 0.05; ** *p* < 0.01; **** *p* < 0.0001.

Given that neopterin is a marker of IFN-γ-mediated immune activation, we evaluated serum IFN-γ levels across groups. Uremic patients exhibited elevated baseline IFN-γ levels compared to healthy controls, with the highest levels observed in patients who subsequently developed acute rejection (Figure 2A). Post-transplantation, IFN-γ levels increased further in AR recipients (Figure 2B), whereas SG recipients showed no significant change (Figure 2C), resulting in significantly higher IFN-γ levels in AR compared to SG at 1 (Figure 2D). By 12 months, IFN-γ levels declined in both groups.

During rejection, ACR exhibited significantly higher IFN-γ levels compared to ABMR (*p* = 0.0076) and mixed rejection (*p* = 0.029) (Figure 2E, Cohen’s f = 0.625, G*power = 0.99). Neopterin levels positively correlated with IFN-γ levels in ACR (Figure 2F), but not in ABMR (Figure 2G) or mixed rejection (Figure 2H). No correlation was observed between the N:C ratio and IFN-γ levels, even within the ACR group (Figure 2I). These results indicate a dominant pro-inflammatory response during cellular rejection and prompted further investigation into the balance between pro- and anti-inflammatory cytokines associated with neopterin levels.

Interleukin-10 (IL-10) is a key anti-inflammatory cytokine known to limit IFN-γ production [33]. We therefore evaluated IL-10 levels and compared the IFN-γ:IL-10 ratio with neopterin levels. Uremic patients exhibited higher IL-10 levels than healthy controls. Post-transplant, IL-10 levels increased further in SG recipients, resulting in significantly higher levels compared to AR (Figure 2J). Within AR, ABMR showed the highest IL-10 levels, although this did not reach statistical significance (Figure 2K, Cohen’s f = 0.4432, G*power = 0.63).

The IFN-γ:IL-10 ratio was highest in ACR and lowest in ABMR (Figure 2L, Cohen’s f = 0.5535, G*power = 0.83), indicating a dominant pro-inflammatory response in cellular rejection. This ratio correlated with neopterin levels in ACR (Figure 2M), but not in ABMR (Figure 2N). A positive but non-significant trend was observed in mixed rejection (Figure 2O).

### 3.8. Immune Cell Population Associated with Neopterin

Neopterin is primarily produced by monocytes and macrophages in response to IFN-γ-dependent T cell interaction [34,35]. To identify cellular correlates, we quantified peripheral immune populations, including CD4+ and CD8+ T cells, NK cells (CD45^+^CD3^−^CD11b^−^CD16/CD56^+^), CD45^+^CD11b^−^CD3^−^CD16/CD56^+^ T cells, and CD3^+^CD4^+^CD25^+^Foxp3^+^ T cells (a Treg-enriched population) using intracellular cytokine staining (ICS) flow cytometry (Figure S1A).

**Figure 3:**
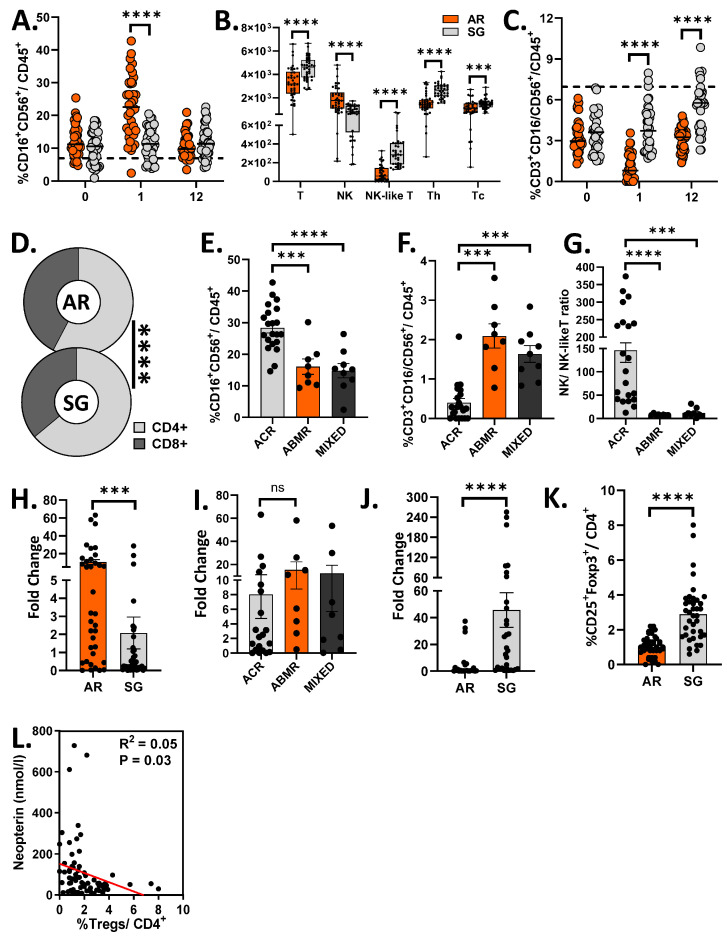
Immunophenotyping of T cells by flow cytometry and mRNA expression of CD80 by real-time PCR. EDTA-anticoagulated peripheral blood samples were collected and processed immediately for both RNA isolation and flow cytometric analysis. For gene expression studies, total RNA was extracted, reverse-transcribed to cDNA, and stored at −80 °C until analysis. On the day of assay, cDNA concentration and purity were assessed, and relative expression of *CD80* and *FOXP3* was quantified using real-time qPCR. For immunophenotyping, PBMCs were isolated by Ficoll density gradient centrifugation, stained with fluorochrome-conjugated antibodies, fixed and acquired on a flow cytometer. (**A**) Frequencies of NK cells in the peripheral blood of AR recipients at pre-transplant, and at 1 and 12 months post-transplant. (**B**) Absolute numbers of T-, NK-, CD3^+^CD16/CD56^+^ T-, Th- and Tc- cells in 1 mL of peripheral blood of AR and SG recipients. (**C**) Frequencies of CD3^+^CD16/CD56^+^ T cells in the peripheral blood of AR recipients. (**D**) Proportions of CD4^+^ Th- cells and CD8^+^ Tc- cells between AR and SG recipients. (**E**–**G**) Frequencies of NK- (**E**) and CD3^+^CD16/CD56^+^ T- (**F**) cells and their ratios (**G**) at the time of ACR, ABMR and mixed rejection. (**H**–**J**) mRNA expression of target gene was quantified in the whole blood by RT-PCR. Peripheral mRNA expression of *CD80* (**H**) and *FOXP3* (**J**) at the time of rejection between AR and SG recipients. Difference in the mRNA expression of *CD80* in different rejection types (**I**). (**K**) Frequencies of circulating CD4^+^CD25^+^Foxp3^+^ T cells between SG at 1 month and AR at the time of rejection. (**L**) Inverse relationship between neopterin and regulatory T cells in transplant recipients. Analysis: Welch’s *t* test ((**A**), B-T/NK, C12, (**D**)); Mann–Whitney U test (B-NKT/Th/Tc, (**H**,**J**,**K**)); Kruskal–Wallis test with Dunn’s multiple comparison test (**F**,**G**,**I**); ordinary one-way ANOVA with Tukey’s multiple comparison test (**E**). ns, not significant; *** *p* < 0.001; **** *p* < 0.0001.

Consistent with our previous findings [25], AR recipients exhibited increased NK cell frequencies and absolute counts compared to SG recipients (Figure 3A,B). In contrast, CD3^+^CD16/CD56^+^ T cells were reduced in AR (Figure 3B,C and Figure S1B) but the proportion of CD8^+^ T cells was significantly higher than the SG group (Figure 3D, 42.23 ± 6.82% vs. 36.14 ± 5.13%, *p* < 0.0001), resulting in a reduced CD4:CD8 ratio (Figure S1C).

NK cell frequencies were significantly higher in ACR than ABMR (Figure 3E, Cohen’s f = 0.955, G*power = 0.99), whereas CD3^+^CD16/CD56^+^ T cells were relatively increased in ABMR (Figure 3F, Cohen’s f = 1.255, G*power = 0.99). Accordingly, the NK-to-CD3^+^CD16/CD56^+^ T cell ratio effectively distinguishes ACR from ABMR (Figure 3G, Cohen’s f = 1.550, G*power = 1).

Macrophages also contribute to the serum IFN-γ pool [36]. However, in this study, we did not specifically characterize macrophages and dendritic cells using cell-specific markers in flow cytometry. Moreover, no significant differences were observed in CD45^+^CD11b^+^ myeloid populations between the AR and SG group (Figure S1C). However, we utilized an indirect approach to evaluate the activation state of these cells during acute rejections by assessing the relative gene expression of *CD80* (*B7.1*) in the peripheral blood using real-time PCR [37]. CD80 mRNA expression was significantly elevated in AR, indicating activation of antigen-presenting cells, including macrophages and dendritic cells (Figure 3H). Although *CD80* expression was higher in ABMR compared to ACR (Fold change, 8.04 ± 3.31 vs. 15.50 ± 6.73), this difference was not statistically significant (Figure 3I, Cohen’s f = 0.1734, G*power = 0.136).

Regulatory T cells (Tregs) represent one of the key sources of IL-10. Therefore, we quantified the peripheral CD4^+^CD25^+^Foxp3^+^ T cell frequencies, acknowledging that this phenotype represents a Treg-enriched population in humans. We found that, in line with the serum IL-10 levels and *FOXP3* gene expression (Figure 3J) [38], and consistent with our previous report [25], CD4^+^CD25^+^FoxP3^+^ T cell frequencies were reduced in AR recipients compared to SG recipients (Figure 3K). These cells negatively correlated with neopterin levels (r = −0.223, *p* = 0.03) (Figure 3L).

Collectively, these findings suggest that the balance between pro-inflammatory and anti-inflammatory immune responses contributes to the regulation of serum neopterin levels during allograft rejection.

Comprehensive data are provided in Tables S2 and S3.

## 4. Discussion

Renal transplantation remains a life-saving intervention for patients with end-stage renal disease, significantly improving survival and quality of life. However, the persistent challenge of organ scarcity and the complexity of diagnosing allograft rejection highlight the need for reliable, minimally invasive biomarkers that can complement existing diagnostic approaches and enable early, mechanism-specific detection of allograft injury. Current monitoring strategies, principally serum creatinine, proteinuria, donor-specific antibody (DSA) testing, and invasive biopsies, are limited by delayed kinetics, lack of specificity, or invasiveness. While biopsy remains the gold standard, its procedural risks, sampling variability, and impracticality for longitudinal monitoring constrain its clinical utility. Consequently, extensive efforts have focused on identifying alternative biomarkers spanning transcriptomic (e.g., CD3ε, IP-10, multigene panels such as KSORT), proteomic (e.g., TNF-α, APOA1), cytokine/chemokine (CXCL9, CXCL10), complement (C4a, sC5b-9), donor-derived cell-free DNA, epigenetic (microRNAs), and cellular immune signatures [39]. Similarly, stable graft-associated B cell gene signatures (e.g., IGKV4-1, MS4A1, TCL1A and others) have provided mechanistic insights into operational tolerance [40]. However, most candidates remain constrained by limited longitudinal validation across heterogeneous populations, dependence on high-throughput technologies requiring specialized expertise, and suboptimal sensitivity or specificity, particularly for early or subclinical immune activation, thereby reinforcing the need for scalable, non-invasive biomarkers adaptable to routine clinical workflows.

In this study, we demonstrate that serum neopterin is a reliable indicator of cellular immune activation during renal allograft rejection. Notably, in our cohort, IFN-γ demonstrated higher diagnostic performance than neopterin in cross-sectional ROC analysis. However, these markers capture biologically distinct aspects of the immune response. IFN-γ represents a proximal, rapidly fluctuating cytokine signal that provides high diagnostic accuracy at the time of active immune activation, but within a limited temporal window. In contrast, neopterin, as a downstream metabolite of IFN-γ-mediated macrophage activation, exhibited a more sustained elevation over time in our longitudinal analysis, including persistence beyond the acute rejection phase. This suggests that neopterin may reflect a temporally integrated measure of immune activation, offering complementary clinical value in settings where the timing of sampling relative to peak immune activity is variable. Accordingly, neopterin should be interpreted not as a replacement for high-performance cytokine markers, but as a stable adjunct biomarker for longitudinal immune monitoring.

In a cohort of biopsy-proved rejection episodes, neopterin achieved 81% sensitivity and 80% specificity at 57 nmol/L, consistent with earlier reports demonstrating elevated neopterin levels during acute rejection and its association with macrophage activation and IFN-γ-driven Th1 responses [41,42,43]. Prior studies across renal and cardiac transplantation have similarly shown that circulating neopterin correlates with rejection severity and immune activation, reinforcing its role as a biomarker of alloimmune response [2].

Neopterin levels stratified rejection phenotypes, with the highest levels observed in ACR, intermediate levels in mixed rejection, and comparatively lower levels in ABMR. This gradient reflects the differential contribution of monocytes, macrophages, T cells, and NK cell-mediated immune responses to neopterin production, in contrast to the antibody-driven mechanisms characteristic of ABMR. Mechanistically, elevated neopterin levels were associated with increased IFN-γ signaling and higher *CD80* mRNA expression, indicative of heightened antigen-presenting cell activation. Furthermore, a decrease in peripheral CD3^+^CD16/CD56^+^ T cells (resulting in an elevated NK-to- CD3^+^CD16/CD56^+^ T ratio) was observed during ACR, while stable grafts and ABMR demonstrated higher CD3^+^CD16/CD56^+^ T cell numbers, which may reflect a more regulatory or less pro-inflammatory immune profile, although the functional properties of this phenotypically defined population cannot be conclusively determined in the present study. The observed reduction in peripheral CD4^+^CD25^+^FoxP3^+^ T cell population and *FOXP3* gene expression, alongside an increased IFN-γ/IL-10 ratio, further supports a shift towards Th1-dominant immunity during cellular rejection. It is important to interpret these findings in the context of human Treg phenotyping limitations. In this study, Tregs were defined as CD4^+^CD25^+^FoxP3^+^ cells, a strategy widely used at the time of data acquisition. However, in humans, FoxP3 expression can be transiently induced in activated conventional CD4^+^ T cells, particularly under inflammatory conditions, and does not necessarily denote stable lineage commitment or suppressive function [44]. The absence of CD127^low/−^ or additional markers therefore limits the ability to definitively distinguish bona fide Tregs from activated effector T cells within this population. Consequently, the observed reduction likely reflects changes in a broader CD4^+^CD25^+^FoxP3^+^ compartment that is enriched for, but not restricted to, regulatory T cells. Importantly, the association of this population with acute cellular rejection remains biologically relevant. However, its precise functional identity should be interpreted with appropriate caution.

In contrast, ABMR, typically diagnosed later than ACR, exhibited a shift from cellular responses to antibody-dependent effector mechanisms, including B-cell activation, plasma cell formation, and antibody production, processes less associated with neopterin secretion. By the time ABMR is diagnosed, cellular immune responses may have diminished, contributing to the relatively lower levels of pro-inflammatory cytokines (reflected in a lower IFN-γ/IL-10 ratio) and neopterin compared to ACR.

From a clinical standpoint, neopterin may offer incremental value when integrated into existing diagnostic workflows. Neopterin is already employed in clinical settings, such as in monitoring immune activation in blood donors in Europe [45]. Unlike biopsy, which is the gold standard but limited by invasiveness, sampling bias, and episodic use, neopterin provides a non-invasive, repeatable measure of immune activation that can be monitored longitudinally. Our findings suggest that neopterin could be integrated into the standard post-transplant monitoring protocols, offering clinicians an additional tool for assessing graft immunological status. Neopterin’s utility may extend beyond rejection diagnostics, potentially enabling early detection of subclinical immune activation and facilitating preemptive therapeutic interventions.

Compared to DSA testing, which is essential for identifying humoral responses in ABMR, neopterin captures early cellular immune activation that may precede or occur independently of antibody formation. This is particularly relevant given that ACR often occurs in the absence of detectable DSA, and delayed recognition can adversely affect graft outcomes. In parallel, conventional inflammatory markers such as CRP and IL-6 lack specificity for alloimmune responses and are frequently confounded by infections or systemic inflammation. In contrast, neopterin is more directly linked to IFN-γ-mediated macrophage activation, providing a more specific reflection of Th1-driven alloimmune processes. In practical terms, neopterin could be incorporated into routine post-transplant surveillance alongside serum creatinine and DSA monitoring, serving as an early “alert” biomarker to trigger closer evaluation or preemptive intervention. Its ease of measurement, cost-effectiveness, and compatibility with standard laboratory assays further enhance its translational potential.

However, an important limitation, also highlighted in prior studies, is that neopterin is not entirely specific to rejection and may be elevated in viral infections or bacterial inflammation [10]. This underscores the need for careful clinical interpretation and supports its use as part of a composite biomarker strategy rather than a standalone diagnostic tool. In this context, integrating neopterin with established modalities, including biopsy findings, DSA profiling, renal function tests, cytokine signatures, and immune cell subset analysis, could enable a more comprehensive and dynamic assessment of graft status. For example, combining neopterin with IFN-γ/IL-10 ratio or NK/NK-Like T cell profiles may improve discrimination between ACR and ABMR, while longitudinal monitoring may help guide the timing of biopsy or optimization of immunosuppressive therapy. In addition, the absence of protocol biopsy confirmation in clinically stable recipients may introduce verification bias, particularly with respect to subclinical rejection, and should be considered when interpreting the diagnostic performance of neopterin.

In conclusion, our findings support neopterin as a biologically relevant and clinically useful biomarker of Th1-mediated immune activation in renal transplantation. While pro-inflammatory cytokines such as IFN-γ may achieve higher diagnostic performance at the time of acute rejection, neopterin provides a more temporally stable and integrative measure of immune activation in our longitudinal analysis. As such, neopterin is best positioned as a complementary biomarker rather than a replacement for biopsy or serological assays. Its integration into current diagnostic frameworks may improve longitudinal monitoring of graft immune status in the context of clinically overt rejection, refine risk stratification, and support more timely and personalized immunosuppressive management. Prospective studies are warranted to further validate its clinical utility and define its role in guiding therapeutic decision-making.

## Figures and Tables

**Table 1:** Demographic details of kidney transplant recipients.

Variables	AR, N = 35	SG, N = 38	*p* Value
Mean age in years (range)	36.34 ± 2.199 (10–58)	33.40 ± 1.567 (12–53)	0.279
Gender (male: female)	29:6	32:6	0.747
Underlying disease for ESRD			0.860
CGN-CRF	14	15	
HTN	9	10	
DM	6	5	
IgA Nephropathy	2	3	
Others	4	5	
Allograft			
LD:CAD	31:4	33:5	0.725
HLA mismatch	3.85 ± 0.2597	3.85 ± 0.2324	1.000
Immunosuppression (induction)			
ATG: Simulect	6:1	8:3	0.102
Immunosupression (maintainance)			
TAC/MMF/steroid	34	36	1.000
CsA/AZA/steroid	1	2	
Ischemic time in minutes			
Warm	5.51 ± 0.33	5.82 ± 0.31	0.495
Cold	79.46 ± 4.20	74.43 ± 4.36	0.409
Potential risk factors			
No. of blood transfusions	5.4 ± 0.5124	5.257 ± 0.462	0.836
No. of dialysis	39.83 ± 3.749	38.63 ± 4.004	0.827
Spousal (H → W) donation	2	3	N/A

Abbreviations: SG: stable graft group; AR: acute rejection group; M: male; F: female; LD: living donor renal allograft; CAD: cadaver donor renal allograft; ATG: antithymoglobulin; TAC: tacrolimus; MMF: mycophenolate mofetil; AZA: azathioprine; CsA: cyclosporine A; N/A: not applicable.

## Data Availability

The original contributions presented in this study are included in the article/Supplementary Material. Further inquiries can be directed to the corresponding author.

## Supplementary Materials

The following supporting information can be downloaded at: https://www.mdpi.com/article/10.3390/biomedicines14040832/s1, Figure S1: Immunophenotyping of T- cells by flowcytometry. A. Representative flow cytometry gating strategy. Lymphocytes were first identified by a forward scatter (FSC) and side scatter (SSC) gate. CD45^+^ cells were co-gated with CD11b^+^ myeloid cells within single cell populations. Non-myeloid CD45^+^ cells were further gated for T-(CD3^+^), NK- (CD16/CD56^+^) and CD3^+^CD16/CD56^+^ T cells. T-helper (Th) cells were identified as CD3^+^CD4^+^ while cytotoxic T (Tc) cells were identified as CD3^+^CD8^+^. B. Frequencies of CD3^+^ T- cells in the peripheral blood of AR recipients at pre-transplant, 1- and 12-months post-transplant period. C. CD4/CD8 ratios in transplant recipients. D. Frequencies of CD11b+ myeloid cells in the peripheral blood of HC, AR and SG recipients; Table S1: Details of antibodies used in the immunophenotyping of PBMCs; Table S2: Comparison between AR and SG at 1-month post-transplantation; Table S3: Comparison among rejection subtypes at the time of rejection.

## Abbreviations

The following abbreviations are used in this manuscript:

CD	Cluster of Differentiation
FoxP3	Forkhead box P3
ICS	Intracellular cytokine staining
IFN	Interferon
IL	Interleukin
mRNA	Messenger RNA

## References

[1] Hoffmann G., Wirleitner B., Fuchs D. (2003). Potential role of immune system activation-associated production of neopterin derivatives in humans. Inflamm. Res..

[2] Murr C., Widner B., Wirleitner B., Fuchs D. (2002). Neopterin as a marker for immune system activation. Curr. Drug Metab..

[3] Haavik J. (1989). From butterflies to neurobiology and the diagnosis of aids. The 100th anniversary of the discovery of pteridines. Tidsskr. Nor. Laegeforen..

[4] Katoh S., Sueoka T., Matsuura S., Sugimoto T. (1989). Biopterin and neopterin in human saliva. Life Sci..

[5] Maerker-Alzer G., Diemer O., Strümper R., Rohe M. (1986). Neopterin production in inflamed knee joints: High levels in synovial fluids. Rheumatol. Int..

[6] Millner M.M., Franthal W., Thalhammer G.H., Berghold A., Aigner R.M., Füger G.F., Reibnegger G. (1998). Neopterin concentrations in cerebrospinal fluid and serum as an aid in differentiating central nervous system and peripheral infections in children. Clin. Chem..

[7] Müller M.M., Curtius H.C., Herold M., Huber C.H. (1991). Neopterin in clinical practice. Clin. Chim. Acta.

[8] Zeimet A.G., Widschwendter M., Knabbe C., Fuchs D., Herold M., Müller-Holzner E., Daxenbichler G., Offner F.A., Dapunt O., Marth C. (1998). Ascitic interleukin-12 is an independent prognostic factor in ovarian cancer. J. Clin. Oncol..

[9] Berdowska A., Zwirska-Korczala K. (2001). Neopterin measurement in clinical diagnosis. J. Clin. Pharm. Ther..

[10] Fuchs D., Weiss G., Reibnegger G., Wachter H. (1992). The role of neopterin as a monitor of cellular immune activation in transplantation, inflammatory, infectious, and malignant diseases. Crit. Rev. Clin. Lab. Sci..

[11] Fuchs D., Weiss G., Wachter H. (1993). Neopterin, biochemistry and clinical use as a marker for cellular immune reactions. Int. Arch. Allergy Immunol..

[12] Niederwieser D., Huber C., Gratwohl A., Bannert P., Fuchs D., Hausen A., Reibnegger G., Speck B., Wachter H. (1984). Neopterin as a new biochemical marker in the clinical monitoring of bone marrow transplant recipients. Transplantation.

[13] Pavletić Z., Labar B., Bogdanić V., Nemet D., Mrsić M., Stavljenić A., Cvoriscec D., Presecki V., Petrovecki M. (1989). Serum neopterin in patients receiving bone marrow transplant. Bone Marrow Transplant..

[14] Volin L., Jansson S.E., Turpeinen U., Pomoell U.M., Ruutu T. (1987). Urinary neopterin in bone marrow recipients. Transplant. Proc..

[15] Bäckman L., Ringdén O., Björkhem I. (1987). Monitoring of serum neopterin levels in renal transplant recipients: Increased values during impaired renal function and cytomegalovirus infection. Nephron.

[16] Carey B.S., Jain R., Adams C.L., Wong K.Y., Shaw S., Tse W.Y., Kaminski E.R. (2013). Serum neopterin as an indicator of increased risk of renal allograft rejection. Transpl. Immunol..

[17] Grebe S.O., Mueller T.F. (2002). Immune monitoring in organ transplantation using neopterin. Curr. Drug Metab..

[18] Myara I., Atger V., Cosson C., Guillemain R., Amrein C., Dreyfus G., Moatti N. (1989). Simultaneous determination of serum neopterin and c-reactive protein as markers of infection in heart-transplant recipients. Clin. Chem..

[19] Jung H.R., Kim M.J., Wee Y.M., Kim J.Y., Choi M.Y., Choi J.Y., Kwon H., Jung J.H., Cho Y.M., Go H. (2019). CD56^+^CD57^+^ infiltrates as the most predominant subset of intragraft natural killer cells in renal transplant biopsies with antibody-mediated rejection. Sci. Rep..

[20] O’Neill M.A., Hidalgo L.G. (2021). NK cells in antibody-mediated rejection—Key effector cells in microvascular graft damage. Int. J. Immunogenet..

[21] Seino K.I., Fukao K., Muramoto K., Yanagisawa K., Takada Y., Kakuta S., Iwakura Y., Van Kaer L., Takeda K., Nakayama T. (2001). Requirement for natural killer T (NKT) cells in the induction of allograft tolerance. Proc. Natl. Acad. Sci. USA.

[22] Hidalgo L.G., Halloran P.F. (2002). Role of IFN-gamma in allograft rejection. Crit. Rev. Immunol..

[23] Khan M.A., Ashoor G.A., Shamma T., Alanazi F., Altuhami A., Kazmi S., Ahmed H.A., Assiri A.M., Broering D.C. (2021). IL-10 mediated immunomodulation limits subepithelial fibrosis and repairs airway epithelium in rejecting airway allografts. Cells.

[24] O’Garra A., Vieira P.L., Vieira P., Goldfeld A.E. (2004). IL-10-producing and naturally occurring CD4^+^ tregs: Limiting collateral damage. J. Clin. Investig..

[25] Dhital R., Minz M., Minz R.W., Nada R., Sharma A., Singh S., Kenwer D., Toki S. (2016). Flow cytometric quantitation of T-cell subsets in peripheral blood of renal allograft recipients. Indian J. Transplant..

[26] Dhital R., Minz M., Minz R.W., Sharma A., Nada R., Singh S., Kenwar D. (2016). Or19 quantification of peripheral B-cell subsets in acute allograft rejection in recipients with renal transplantation. Hum. Immunol..

[27] Minz M., Dhital R., Minz R., Sharma A., Nada R., Singh S., Kenwar D. (2016). mRNA expression of BAFF and APRIL receptors increases in acute rejection in kidney transplant recipients. Am. J. Transplant..

[28] Minz M., Dhital R., Minz R., Sharma A., Nada R., Singh S., Kenwar D. (2016). Decreased number of plasma cells in peripheral circulation is associated with acute rejection in renal transplantation. Transplantation.

[29] Minz M., Dhital R., Minz R., Sharma A., Nada R., Singh S., Kenwar D. (2016). Increased gene expression of BAFF-R, TACI and BCMA is associated with acute rejection in kidney transplantation. Transplantation.

[30] Minz M., Dhital R., Minz R., Sharma A., Nada R., Singh S., Kenwar D. (2016). Post-transplant reduction of long lived plasma cells from peripheral circulation is associated with acute rejection in kidney transplant recipients. Am. J. Transplant..

[31] Dhital R., Minz M., Minz R.W., Sharma A., Singh S., Kenwerr D., Kumar S. (2016). Peripheral lymphocytes and immunoglobulins in patients with end stage renal disease: A single institutional experience. Indian J. Nephrol..

[32] Haas M., Sis B., Racusen L.C., Solez K., Glotz D., Colvin R.B., Castro M.C.R., David D.S.R., David-Neto E., Bagnasco S.M. (2014). Banff 2013 meeting report: Inclusion of c4d-negative antibody-mediated rejection and antibody-associated arterial lesions. Am. J. Transplant..

[33] D’Andrea A., Aste-Amezaga M., Valiante N.M., Ma X., Kubin M., Trinchieri G. (1993). Interleukin 10 (IL-10) inhibits human lymphocyte interferon gamma-production by suppressing natural killer cell stimulatory factor/IL-12 synthesis in accessory cells. J. Exp. Med..

[34] Barak M., Merzbach D., Gruener N. (1989). Neopterin measured in serum and tissue culture supernates by a competitive enzyme-linked immunosorbant assay. Clin. Chem..

[35] Wirleitner B., Reider D., Ebner S., Böck G., Widner B., Jaeger M., Schennach H., Romani N., Fuchs D. (2002). Monocyte-derived dendritic cells release neopterin. J. Leukoc. Biol..

[36] Darwich L., Coma G., Peña R., Bellido R., Blanco E.J.J., Este J.A., Borras F.E., Clotet B., Ruiz L., Rosell A. (2009). Secretion of interferon-gamma by human macrophages demonstrated at the single-cell level after costimulation with interleukin (IL)-12 plus IL-18. Immunology.

[37] Mir M.A., Mir M.A. (2015). Concept of reverse costimulation and its role in diseases. Developing Costimulatory Molecules for Immunotherapy of Diseases.

[38] Dhital R., Anand S., Graber B., Zeng Q., Velazquez V.M., Boddeda S.R., Fitch J.R., Minz R.W., Minz M., Sharma A. (2022). Murine cytomegalovirus promotes renal allograft inflammation via Th1/17 cells and IL-17A. Am. J. Transplant..

[39] Minz R.W., Singh J., Kenwar D.B., Pattanaik S., Sharma A. (2026). Chapter 8—Biomarkers of renal transplant rejection: Yesterday, today, and tomorrow. Biomarkers in Kidney Transplantation.

[40] Dhital R., Sigdel T.K., Pattanaik S., Sharma A. (2026). Chapter 11—Biomarkers of tolerance in renal transplantation. Biomarkers in Kidney Transplantation.

[41] Kameoka H., Takahara S., Takano Y., Moutabarrik A., Kokado Y., Ishibashi M., Sonoda T., Okuyama A. (1994). Serum and urinary neopterin as markers in renal transplant patients. Int. Urol. Nephrol..

[42] Schäfer A.J., Daniel V., Dreikorn K., Opelz G. (1986). Assessment of plasma neopterin in clinical kidney transplantation. Transplantation.

[43] Wolf J., Musch E., Neuss H., Klehr U. (1987). Neopterin in the serum and urine in the differential diagnosis of disorders of kidney function following kidney transplantation. Klin. Wochenschr..

[44] Allan S.E., Crome S.Q., Crellin N.K., Passerini L., Steiner T.S., Bacchetta R., Roncarolo M.G., Levings M.K. (2007). Activation-induced FOXP3 in human t effector cells does not suppress proliferation or cytokine production. Int. Immunol..

[45] Hönlinger M., Fuchs D., Hausen A., Reibnegger G., Schönitzer D., Werner E.R., Reissigl H., Dierich M.P., Wachter H. (1989). Serum-neopterinbestimmung zur zusätzlichen sicherung der bluttransfusion: Erfahrungen an 76,587 blutspendern. DMW—Dtsch. Med. Wochenschr..

