# Supplementary material for: Neopterin as a Biomarker of Cellular Immune Response in Renal Allograft Rejection Subtypes: Linking Cytokines and Immune Cells to Improve Diagnostic and Therapeutic Approaches
Source: Biomedicines. 2026 Apr 6;14(4):832. doi: 10.3390/biomedicines14040832 (PMC13113380; doi:10.3390/biomedicines14040832)
Supplement: Supplementary file 1 [file biomedicines-14-00832-s001.zip › biomedicines-4177675-supplementary.pdf]

SUPPLEMENTARY FILE

Title: Neopterin as a biomarker of cellular immune response in renal allograft rejection subtypes: Linking cytokines and immune cells to improve diagnostic and therapeutic approaches.

Authors: Ravi Dhital, Ph.D., Mukut Minz, M.S., Ranjana W. Minz, M.D., Shashi Anand, M.Sc., Ritambhra Nada, M.D., Sarbpreet Singh, M.S., Deepesh B. Kenwar, M.S., Ashish Sharma, M.S.

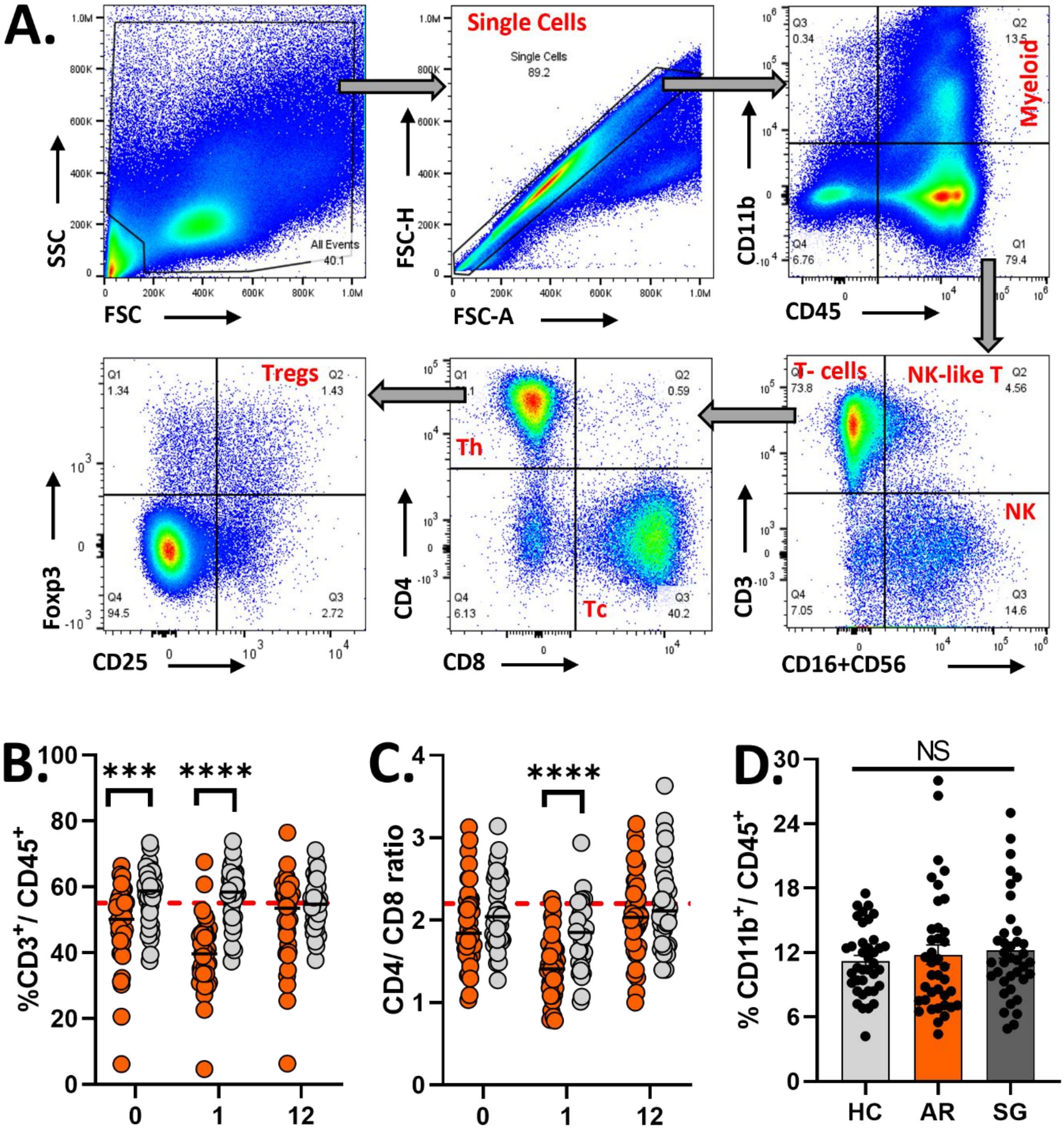

Figure S1. Immunophenotyping of T- cells by flowcytometry. A. Representative flow cytometry gating strategy. Lymphocytes were first identified by a forward scatter (FSC) and side scatter (SSC) gate. $CD45^+$ cells were co-gated with $CD11b^+$ myeloid cells within single cell populations. Non-myeloid $CD45^+$ cells were further gated for T- ($CD3^+$), NK- ($CD16/CD56^+$) and $CD3^+CD16/CD56^+$ T cells. T-helper (Th) cells were identified as $CD3^+CD4^+$ while cytotoxic T (Tc) cells were identified as $CD3^+CD8^+$. **B.** Frequencies of $CD3^+$ T- cells in the peripheral blood of AR recipients at pre-transplant, 1- and 12- months post-transplant period. **C.** CD4/CD8 ratios in transplant recipients. **D.** Frequencies of $CD11b^+$ myeloid cells in the peripheral blood of HC, AR and SG recipients.

Table S1. Details of antibodies used in the immunophenotyping of PBMCs.

Target	Conjugate	Manufacturer	Clone	Catalogue #	Dilution
CD16	FITC	BD	NKP15	347523	1 in 200
CD56	FITC	BD	NCAM16.2	340410	1 in 200
FoxP3	PE	BD	236A/E7	560852	1 in 300
CD45	PerCp-Cy5.5	BD	2D1	340953	1 in 50
CD4	PE-Texas Red	ThermoFisher	S3.5	MHCD0417	1 in 25
CD11b	PE-Cy7	BD	ICRF44	557743	1 in 50
CD8	APC	BD	RPA-T8	561952	1 in 250
CD25	APC-Cy7	BD	M-A251	557753	1 in 100
CD3	Pacific Blue	BD	UCHT1	558117	1 in 100

Table S2. Comparison between AR and SG at 1-month post-transplantation

Markers	AR group (N=38)	SG group (N=38)	P-value
Neopterin (nmol/l)	156.20 ± 28.34	55.86 ± 9.59	0.0018
IL-10 (pg/ml)	7.88 ± 0.56	13.66 ± 0.69	<0.0001
IFN-Y (pg/ml)	21.34 ± 2.08	1.90 ± 0.19	<0.0001
IFN-Y: IL-10	3.56 ± 0.50	0.15 ± 0.01	<0.0001
NK cells (%/ CD45+)	22.53 ± 1.52	11.35 ± 0.78	<0.0001
NKT cells (%/ CD45+)	1.70 ± 0.25	5.28 ± 0.36	<0.0001
NK: NKT	85.21 ± 18.03	3.69 ± 0.39	<0.0001
mRNAFoxP3 (FC)	5.25 ± 2.22	45 ± 12.95	0.0080
Tregs (%/ CD4+)	1.07 ± 0.09	2.95 ± 0.26	<0.0001
mRNACD80 (FC)	10.69 ± 2.76	2.07 ± 0.88	0.0034

FC=Fold Change.

Table S3. Comparison among rejection subtypes at the time of rejection.

Markers	ACR (N=21)	ABMR (N=8)	Mixed (N=9)	p-value
Neopterin (nmol/l)	199.2 ± 39.71	58.59 ± 12.42	142.7 ± 68.84	NS
IL-10 (pg/ml)	8.24 ± 0.83	9.30 ± 0.73	5.77 ± 0.90	0.0490
IFN-Y (pg/ml)	26.98 ± 2.79	12.19 ± 3.51	16.33 ± 2.77	0.0055
IFN-Y: IL-10	4.12 ± 0.60	1.44 ± 0.51	3.16 ± 0.41	0.0273
NK cells (%/ CD45+)	28.31 ± 1.56	16.08 ± 2.43	14.78 ± 2.23	<0.0001
NKT cells (%/ CD45+)	0.40 ± 0.10	2.09 ± 0.30	1.63 ± 0.21	<0.0001
NK: NKT	146.0 ± 25.93	8.00 ± 0.67	11.92 ± 3.20	0.0002
Tregs (%/ CD4+)	0.84 ± 0.13	1.43 ± 0.12	1.28 ± 0.17	0.0244
